# The risk factors, consequences, treatment, and importance of gestational depression

**DOI:** 10.4274/tjod.42744

**Published:** 2015-06-15

**Authors:** Elif Akkaş Yılmaz, Çağrı Gülümser

**Affiliations:** 1 Sami Ulus Women and Children’s Diseases Training and Research Hospital, Clinic of Obstetrics and Gynecology, Ankara, Turkey; 2 Başkent University Faculty of Medicine, Department of Obstetrics and Gynecology, Ankara, Turkey

**Keywords:** Pregnancy, depression, complications, risk factors, treatment

## Abstract

Nowadays, mental problems have become an important health issue, the most frequent of which in pregnancy is depression. Gestational depression is known to increase gestational complications and negatively affect maternal and fetal health. The frequency of gestational depression and depressive symptoms are 10-30%. Risk factors vary according to genetic, psychologic, environmental, social, and biologic factors. Maternal morbidity and mortality rates increase in pregnant women who do not receive treatment, obstetric complications and negative fetal consequences are seen, and the incidence of postpartum depression increases. Due to all these important consequences, healthcare providers who manage pregnant women should be informed about the frequency, symptoms, and screening methods of postpartum depression, the significance of the consequences of undiagnosed and untreated depression on the health of mother and baby, and the importance of early diagnosis. Pregnant women who are at risk should be screened and detected, and directed to related centers. In this review, we briefly review the definition of gestational depression, its frequency, risk factors, complications, screening, treatments, and the procedures that need to be performed the diagnostic process.

## INTRODUCTION

Today, the incidence of mental problems are significantly increased, and have become both an individual and a public issue. Of all the mental disorders, the most frequent and the one that carries the greatest burden is depression. Depression is an illness that decreases onereaquality of life by making functions, creativity, happiness, and satisfaction fade away and reduces the capacity for work. Its frequency, chronicity, high rates of recurrence, and suicide incidence makes it an important health issue and the third most important disease in the world in terms of its burden. Depression is ranked as the eighth greatest healthcare burden in low-income countries; however, it is number one in countries that have average and higher incomes^(1,2)^. Depression is the most frequent of all mental disorders in the gestational period. In past years, the gestational period was known to bring a sensation of well-being and this was thought to protect against mental disorders. However, today it has been recognized that gestational depression has been missed because the physiologic changes and symptoms seen in depression are similar to those in the gestational and postpartum period (e.g. sleeping habits, appetite, weight changes, and fatigue)^(3)^. According to World Health Organizationes, pression hurden ncrea^(4)^, 1 in 3-5 pregnant women in developing countries, and 1 in 10 pregnant women in developed countries have severe mental problems either during gestation or in the postpartum period. Depression in pregnancy is the most important risk factor for postpartum complications and fetal-neonatal problems, and is more frequent than in the postpartum period. Gestational depression has become an important issue. Despite it being such an important health issue, the development of depression during pregnancy is not being detected and consequently women are not receiving proper treatment because there is still no screening program and healthcare providers are not yet sufficiently knowledgeable. Here, we briefly review, the definition of gestational depression, its frequency, risk factors, symptoms, risks for mother and baby, screening, and treatment.

## THE PREVALENCE OF GESTATIONAL DEPRESSION IN THE WORLD AND IN TURKEY

According to the literature, the frequency of gestational depression and depressive symptoms is between 10-30%^(5,6,7,8,9)^. In the study of Bödecs et al. performed in Hungary^(10)^, the prevalence of gestational depression was found to be 18%, the result of the Marcus et al. study in the United States of America was 20%, and Kurki et al. reported 30% in Finland^(11,12)^. In the studies in our country, Turkey, the gestational depression prevalence has been reported to be between 27.9-33.1%^(13,14,15,16,17)^. As these numbers are higher than the values of the world, it is estimated that factors in the lives of pregnant women in our country affect the prevalence and intensity of the gestational depression (e.g. teenage pregnancies, frequent pregnancies, economic problems, low education levels, violence in the family, crowded families)^(14,15)^.

## RISK FACTORS OF GESTATIONEL DEPRESSION

As shown in Table 1, the potential risk factors of gestational depression should be considered through different perspectives. In the literature, the affects of genetic, psychologic, environmental, social and biologic factors are considered. History of depression, history of depression in the family, marital problems, lack of partner, lack of social support, negative experiences, violence, lower social and economic levels, poor obstetric history (abortus, death), unwilling pregnancies, very early or late pregnancies, and low education levels form the risk factors and these vary according to cultures^(6,7,18,19,20)^. In a randomized study conducted by Leigh et al. in Australia on 367 pregnant women^(21)^, depression risk was found to be high in those who lacked self-respect, were anxious in pregnancy, lacked social support, had lower incomes, and those who experienced a significant trauma. In the study of Figueiredo et al. performed in Portugal, teenaged pregnant girls showed significantly more depressive symptoms during their pregnancies and postpartum periods^(22)^. Those who receive antidepressants are under the risk of depression and its recurrence when they terminate their medication after getting pregnant; the first 8 weeks after termination of medication carry the greatest risk. Cohen et al. found that relapses of depression occurred in 43% women who had had a major attack of depression, 26% of those who continued their medication, and 68% of those who terminated their medication depression^(23)^. In most cases, miscommunication, unwilling pregnancies, and marital problems are more frequent. All complications seen in pregnancy and all the medical problems that make a pregnancy risky have the potential to cause psychiatric symptoms. The incidence of anxiety and depression is higher in the gestational- and postpartum period in pregnant women who have medical problems like hypertension and diabetes, and women who have obstetric problems like preeclampsia, risk of early labor, poli/oligohidramnios, and intrauterine growth retardation when compared with those without medical problems^(24,25)^.

## FINDINGS AND SYMPTOMS OF GESTATIONAL DEPRESSION

It can frequently be hard to diagnose gestational depression because the findings and symptoms of depression in pregnant women are so similar to the physiologic changes and symptoms during pregnancy. The major findings and symptoms of depressed mood during pregnancies are; changes in sleep habits and appetite, pain, fluctuation in sensations, abnormal fatigue, lack of libido and concentration, and anxiety and fears about delivery. Even if these symptoms of depression cannot be separated from the symptoms of general depression, somatic symptoms like nausea, stomach ache, tachypnea, hyperpnoea, headache, gastrointestinal symptoms, tachycardia, and lightheadedness are significantly more likely to be seen, hyperactive physical symptoms have to be important.

## THE AFFECTS AND CONSEQUENCES OD THE GESTATIONAL DEPRESSION

The consequences of gestational depression can be seen in Table 2, grouped as maternal, fetal, and childhood originated. Maternal morbidity, mortality, and suicide attempt rates increase in women who have depression during pregnancy and do not receive treatment. Gestational depression affects both the physical and mental health of women by decreasing their self-hygiene and increasing gestational complications, which negatively effect the health of the fetus. Preeclampsia and eclampsia, early-onset labor, babies with lower weight, and APGAR have been found to be more related to the depression during pregnancy^(12,26,27)^. If no is action taken during pregnancy and depression continues, the risk increases in the babies and children. These negative effects present as problems in bond development between babies and mothers, growth retardations, development of motor and linguistic skills, and increased risk in gastrointestinal and lower respiratory infections^(28,29,30)^. Sensory and cognitive problems are known to develop in such children in later years^(31,32)^. Many studies have proven that gestational depression is the most important risk factor of postpartum depression and depression continues during the postpartum period in 50% of women who had depression during their pregnancies^(21,33,34)^.

## EFFECTS OF GESTATIONAL DEPRESSION ON PREGNANT WOMEN

Gestational depression decreases mothers’ self-hygiene, which can harm cognitive functions in terms of decision-making ability, this situation may be correlated with lack of concentration during pregnancy and use addictive substances. Many studies have shown that women with gestational depression use tobacco and alcohol and addictive substances during their pregnancies^(35,36)^. In the study of Zuckerman et al., depression in pregnancy was highly correlated with tobacco, alcohol, and cocaine use^(37)^. Pregnant women with symptoms of depression are more likely to miss their screenings, tend to receive less medical help, and have less pre-delivery help. These patients generally have nutritional and sleeping problems and gain less weight than the normal because of their loss of appetite. It is known that those who have depression have decreased social function, become introvert, and have fears about becoming parents. In patients for whom depression continues after delivery, provision of reduced care, increased anxiety, and thoughts about harming offspring can be seen. Severe depression may lead to self-harm, an increased risk of showing brutal actions, and may cause suicide^(38,39,40,41)^. Hesse et al. showed that 5% of patients who had depression during pregnancies and received no treatment had attempted suicide^(42)^. Of the causes of death directly connected to pregnancy, suicide accounts for 2.4% of deaths related to pregnancy, and 3.2% of maternal deaths.

## SCREENING OF GESTATIONAL DEPRESSION

It is vital to diagnose depression in pregnant women early because it is important to give medication to decrease long-term negative consequences. The American College of Obstetricians and Gynecologists Committee advises that all women, regardless of social status, education level, and ethnicity, should be screened at least once every trimester for mental disorders^(43)^. The screening should be done using short, reliable, valid methods that have high sensitivity and low false positive rates. The commonly-used methods for the screening of depression during pregnancy include the Patient Health Questionnaire, Beck Depression Inventory, Center for Epidemiologic Studies Depression Scale, Two-Sentence test, and the Edinburgh Postpartum Depression Scale^(44,45,46)^. Supplements 1,2,3,4 demonstrate all screening tools mentioned above for gestational depression.

## TREATMENT APPROACHES IN GESTATIONAL DEPRESSION

The treatment of gestational depression is becoming an important issue for researchers and physicians. In the last few years, physicianss studies have mostly been about concerns of medications used in the treatment of depression and how they effect the fetus; however, now it is understood that the real problem is depression without medication. Today, the view is that gestational depression should be treated because of its negative consequences on both mother and fetus. There are 3 problems that clinicians have to solve when they meet a mother in depression:

Women who become pregnant while using antidepressants and continue using them for some time without being aware that they are now pregnant: according to todaya mother in dethis situation carries low risk; however, a conversation with the mother and relatives should take place to provide information about the risks and a decision about the pregnancy has to be made.

Depression began before pregnancy and is ongoing or the depression occurred during pregnancy: For women who have not been given medication, if psychotherapy cannot be undertaken or is insufficient, treatment especially with an SSRI is the most accepted approach that is accepted the most because the risk of damage to mother and baby is high.

Babies with possible withdrawal symptoms whose mother had medical treatment during pregnancy: Most of these symptoms can be cured by general support but it is important for physicians to be aware of the situation and closely observe the baby. Patients and their relatives have to be involved in all decision processes. It is extremely difficult to form a treatment method that can be applied to all pregnant women. It is the responsibility of the physician to evaluate all cases on an individual basis and form a treatment strategy for the sake of mother and child.

## PSYCHOTHERAPY

Psychotherapies are the first choice in the treatment of depression because of the possible adverse effects of medical therapy, especially in mild and moderate depression (for patients who do not have recurrent depressive episodes, severe weight loss, suicide attempts and who are not inpatients) (Grade 2B). Short-term physiotherapies in particular have been found to be effective and they are being used more frequently. Interpersonal and cognitive behavioral therapies are reported to be efficient for mild and moderate depression.

## INTERPERSONEL PSYCHOTHERAPIES

Interpersonal physiotherapies are short therapies that focus on interpersonal problems that aim to decrease depressive symptoms and fix interpersonal functions. They also serve to help form social support systems that will help to cope with the stress. Acute interpersonal problems can be discussed in such an environment; cognitive processes and previous relations are not addressed in this way. Treatment time is between 12-20 weeks, once a week^(47,48,49)^. It has been shown to be effective for both gestational and postpartum depression. On the study reported on 120 women who had major depression and had given birth the women were separated into 2 randomized groups, one of which received interpersonal psychotherapy for 12 weeks^(50)^. The authors found a significant difference in terms of remission for the group that received physiotherapy (37.5%) compared with the con-treatment group (13.7%).

## COGNITIVE BEHAVIORAL THERAPY

Cognitive behavioral therapy is one of the most commonly-used psychotherapy methods of the world, and is effective for most mental disorders. This therapy aims to fix depression by modifying schemes formed by cognitive distortions, which effect emotions and result in depression. Approximately 12 sessions are needed for depression.

## MEDICAL TREATMENTS

Medical treatment should be considered when psychotherapies are difficult to perform or become unsuccessful, in cases of moderate depression, history of severe depression, positive outcomes of medical treatments, and the possibility of the mothern, positive outcomes high. Even though the reliability of these medications not yet proved due to the difficulty of performing studies on pregnant women, according to the outcomes of the actual medications, there is no major malformations affect in fetuses. Before starting a medication in pregnant women, risks for a developing fetus must be evaluated in terms of malformations of organs, teratogenesis, neonatal withdrawal and toxicity syndromes, and long-term behavioral effects. Before initiating a medication, the potential harms and benefits and severity of depression should be considered^(51,52)^. The treatment of gestational depression is summarized in Table 3.

SSRIs: SSRIs are the first-choice medications according to the literature (Grade 2B). They are a group C medication for pregnant women. The first medication of this group is fluoxetine and it is the most well known for use in pregnancy. No evidence of teratogenity in the children women who used fluoxetine, fluvoxamine, sertraline, and paroxetine during their pregnancies has been reported in the literature; however, some medical problems have occurred^(53,54)^. According to some studies, early onset of labor and lower weight of the baby, lower APGAR scores and persistent pulmonary hypertension in the neonate have occurred when used in the late periods of pregnancy^(53,54)^. When compared with other antidepressants, paroxetine was reported to lead to more congenital malformations, especially cardiac abnormalities, although the studies may have had conflictions; there is no definite conclusion^(55,56,57)^. Neonates who are exposed to SSRIs in the third trimester can display symptoms of withdrawal (irritability, hypotonia, mild respiratory problems, and eating and sleeping disorders) but these can be easily treated with general support. Still, for the initial therapy, sertraline is the most recommended; citalopram is also an appropriate alternative.

For women with severe depression who do not respond to SSRIs, venlafaxine and bupropion should be chosen instead of other antidepressants (Grade 2C). In addition, electroconvulsive therapy can be considered.

**Tricyclic Antidepressants:** Even though there are differences between studies in the literature, the general consensus is that there is no increase in the risk of congenital malformations in neonates who are exposed to tricyclic antidepressants in the first trimester when compared with the general population. Functional intestinal obstruction and urinary retention can be seen in neonates due to anti-cholinergic adverse effects with all the tricyclic antidepressants. especially with clomipramine^(58,59,60,61,62,63,64)^.

**Electroconvulsive Therapy (ECT):** Electroconvulsive therapy is recommended in the event that patients do not respond to psychotherapy and recurrent medical treatments (3-5 times). Especially in the major depression of the pregnant women, it is accepted as fast, reliable, and safe. In the literature, although some studies have reported fetal death, decrease in fetal heart rate, increase in uterine contractions, and early onset of labor, ECT is accepted today as an effective way to treat severe depression during pregnancy and its risks are minimal for mother and fetus^(65,66,67)^.

## RESULTS

Mother and child health is one of the most important subjects of the World Health Oraganization’ a expectations for 2015 and for the Millennium Development Plans. In the past, even if the focus was only on the physical health of mother and child and mental health was ignored, today, the findings show that their physical and mental health cannot be separated and progress can be made only with them being combined. For this reason, knowing the risk factors of gestational and postpartum depression, and closely screening those at risk are important. It should be remembered that early diagnosis and treatment has positive effects on the physical and mental health of the mother and baby and their relationship^(68)^.

Gestational depression is as important as postpartum depression and should be diagnosed and treated early; however, it has not been considered as an important issue. Despite the many studies on gestational depression, only recently has the issue become important. Pregnancy is difficult period in terms of mental status and as yet, no standard for psychiatric support exists in Turkey even though gestational depression and anxiety are acknowledged to be very frequent, and importance is given to preterm care. Women with gestational depression are often not examined and consequently go untreated. When the effects on baby and mother are considered, women who are at risk of depression and anxiety must be identified early in healthcare centers, and should receive follow-up and treatment as required. During pregnancy, women should undergo the necessary assessments to maintain their physical and mental well-being, and these assessments ought to become a part of regular follow-ups. Healthcare providers who manage pregnant women should be educated about the frequency, symptoms, and screening methods of gestational and postpartum depression, the effects on mother and babyhs health if depression goes undiagnosed and untreated. In addition, clinicians must be informed about the risk factors of postpartum depression and the importance of watching closely for those exposed to these risks. Finally, depression-screening programs should be formed in Turkey in which pregnant women are seen by professional groups in order to detect and prevent mental disorders, like they are in developed countries. Those who are detected as being at risk as a result of these screening programs should be referred for necessary treatment at appropriate centers.

## Figures and Tables

**Supplement 1:**
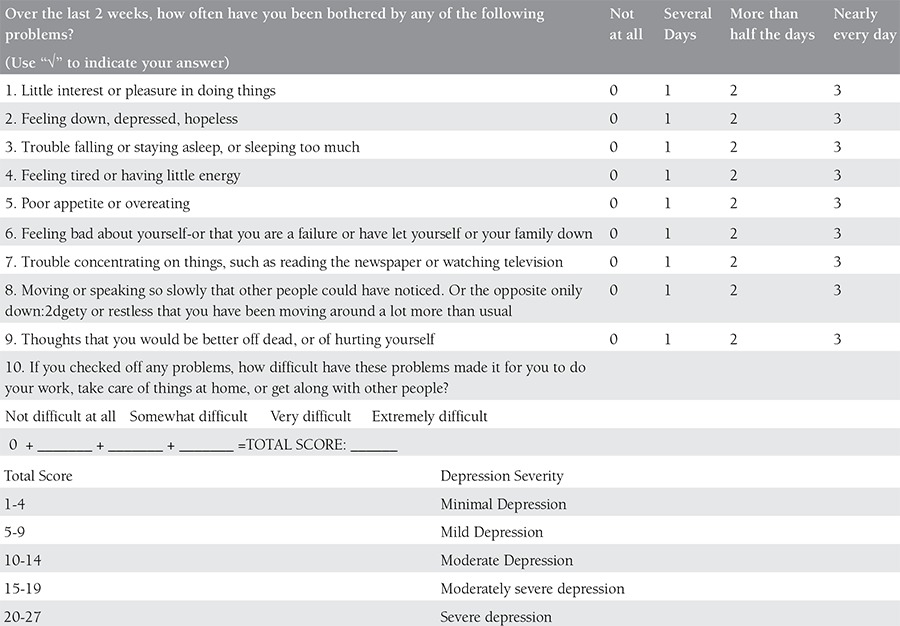
Patient Health Questionnaire (PHQ-9)

**Supplement 2:**
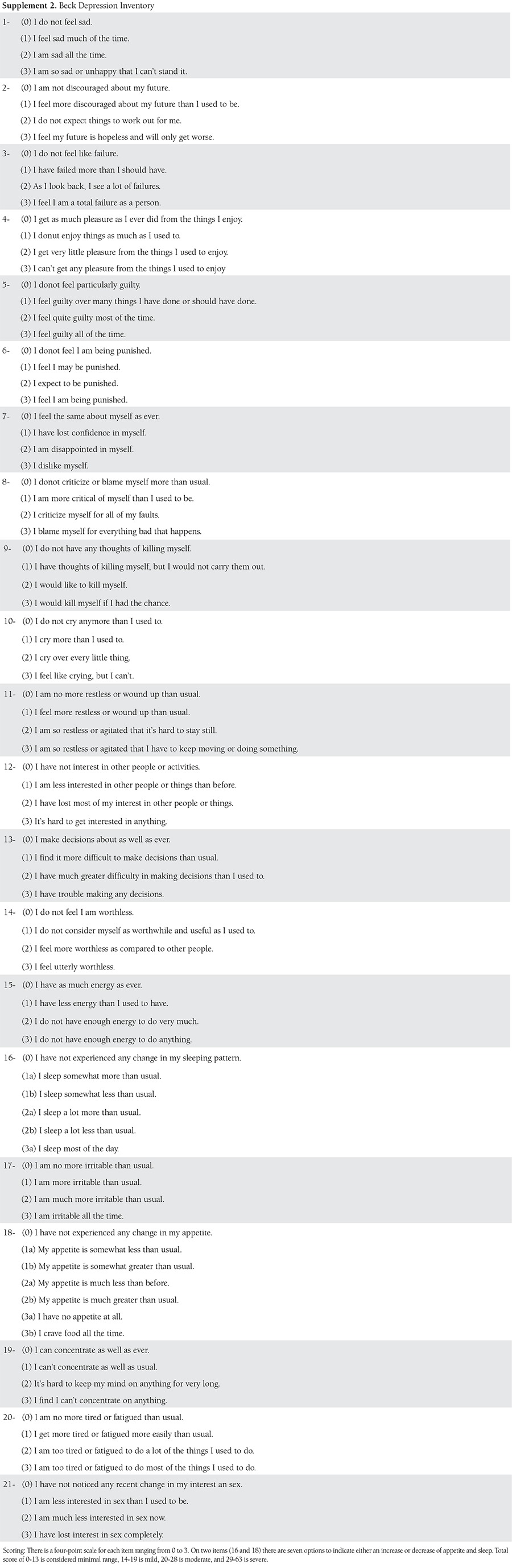
Beck Depression Inventory

**Supplement 3:**
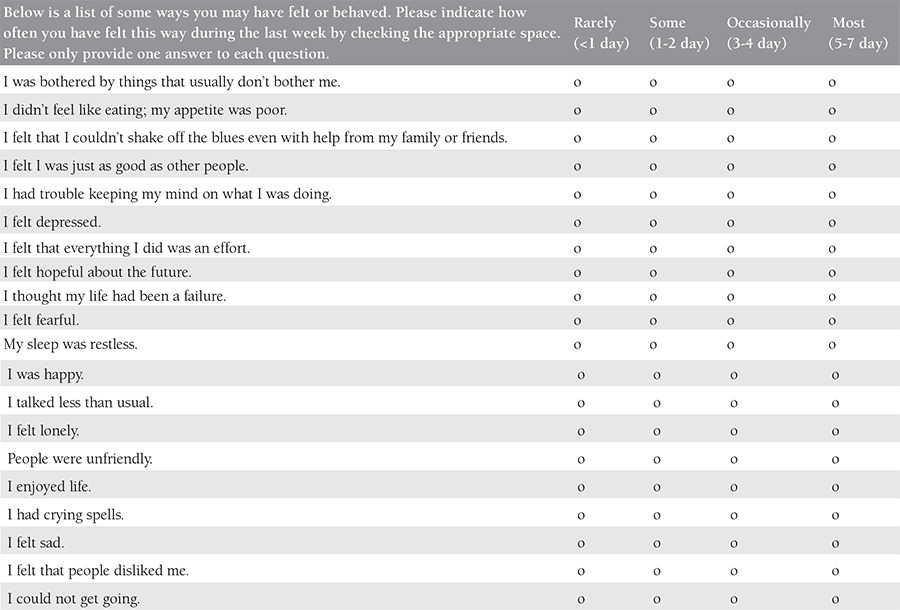
Center for Epidemiologic Studies Depression (CES-D)

**Supplement 4:**
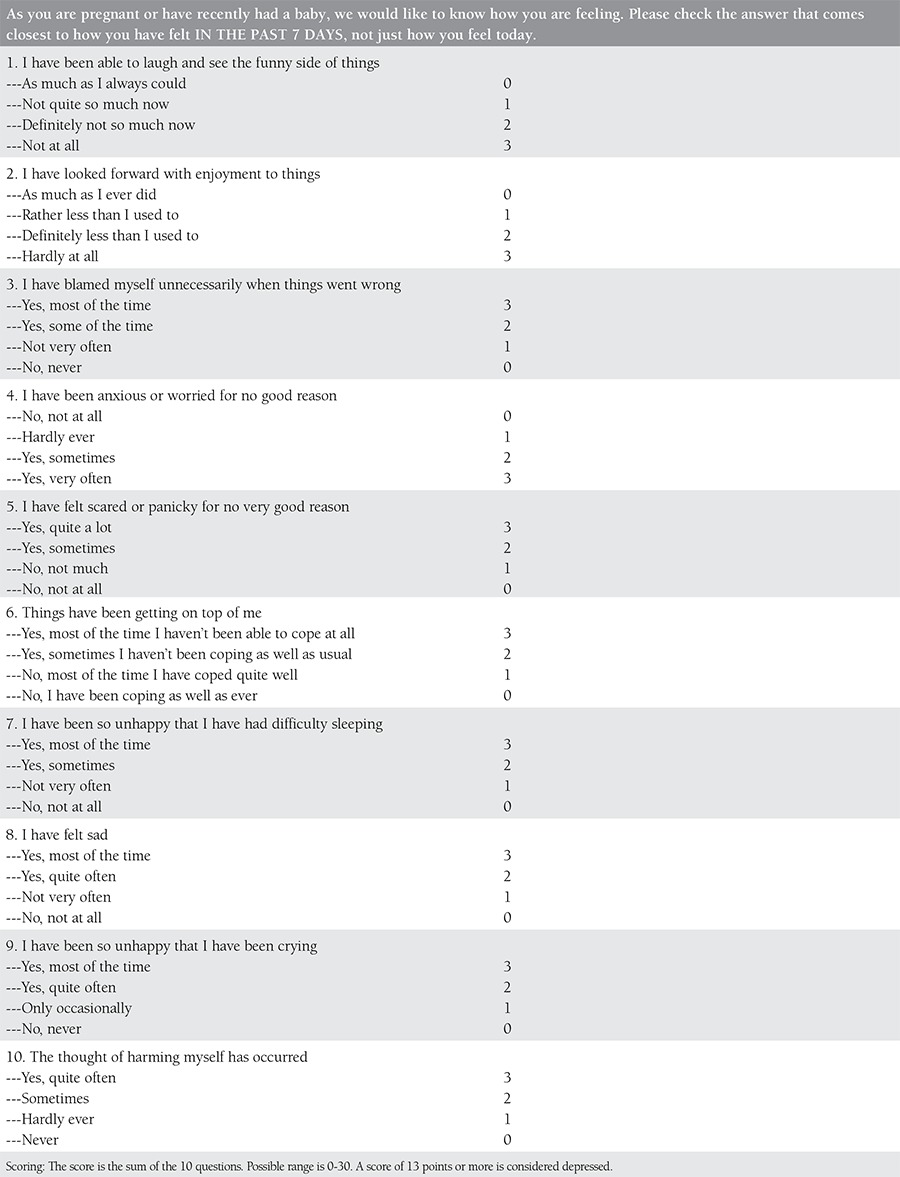
Edinburgh Postnatal Depression Scale (EPDS)

**Table 1:**
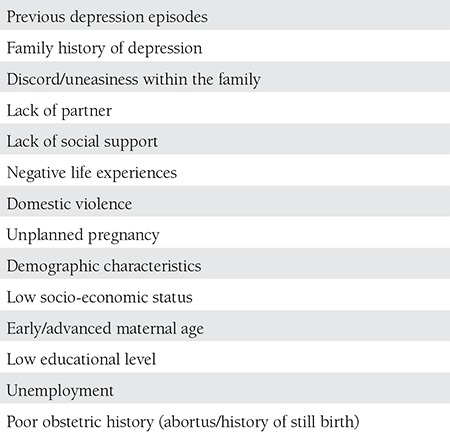
Risk factors for antepartum depression

**Table 2:**
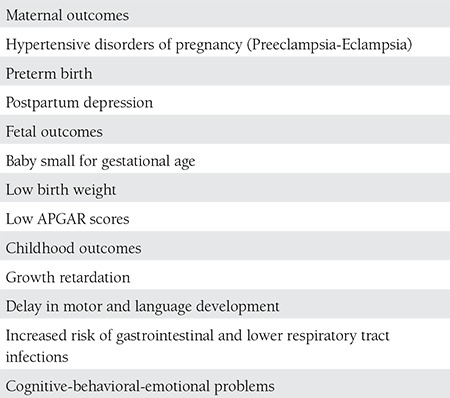
Outcomes of antenatal depression

**Table 3:**
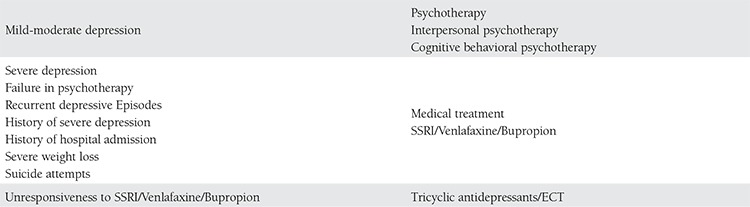
Treatment of antepartum depression
